# Effects of temperature and environmental covariates on the dynamic transmission of hand, foot, and mouth disease in Zhejiang, China

**DOI:** 10.1371/journal.pntd.0012884

**Published:** 2025-03-18

**Authors:** Wanqi Wen, Ziping Miao, Dashan Zheng, Feng Ling, Zhengmin (Min) Qian, Benjamin de Foy, Steven W. Howard, Jimin Sun, Hualiang Lin

**Affiliations:** 1 Department of Epidemiology, School of Public Health, Sun Yat-sen University, Guangzhou, Guangdong, China; 2 Key Laboratory of Vaccine, Prevention and Control of Infectious Disease of Zhejiang Province, Zhejiang Provincial Center for Disease Control and Prevention, Hangzhou, Zhejiang, China; 3 Department of Epidemiology and Biostatistics, College for Public Health and Social Justice, Saint Louis University, Saint Louis, Michigan, USA; 4 Department of Earth and Atmospheric Sciences, School of Science and Engineering, Saint Louis University, Saint Louis, Michigan, USA; 5 Department of Health Services Administration, School of Health Professions, University of Alabama at Birmingham, Birmingham, Alabama, USA; University of California San Diego, UNITED STATES OF AMERICA

## Abstract

**Background:**

Studies have documented the impact of temperature on the incidence of hand, foot, and mouth disease (HFMD); however, no study has examined its impact on the transmissibility.

**Methods:**

The longitudinal surveillance data of HFMD in Zhejiang Province during 2013-2019 were collected from National Notifiable Infectious Diseases Reporting Information System. The incidence of HFMD was represented by daily case counts, and the transmissibility was quantified as the instantaneous reproductive number ($R_{t}$). The case time series design was applied to investigate the association between temperature and HFMD incidence at small-scale spatial patterns (i.e., townships). General additive model was further employed to analyze the effects of temperature and other driving factors on the transmissibility of HFMD. Separate models were also conducted for each city, along with seasonal and spatial stratified analysis.

**Results:**

We observed an inverted V-shaped association between temperature and HFMD incidence, with the highest cumulative relative risk (RR: 3.81, 95% CI: 3.75-3.86) at 28°C compared to the reference temperature. Notably, we discovered that HFMD transmissibility exhibited a similar but more pronounced sensitivity to temperature changes, peaking at a lower temperature of 19.69°C. City-specific and stratified results were aligned with the overall provincial pattern. Additionally, other significant driving factors of HFMD transmissibility included the depletion of susceptible individuals, school holidays, vaccination program, relative humidity, and the Normalized Difference Vegetation Index.

**Conclusion:**

Nonlinear associations between temperature and HFMD incidence, as well as transmissibility, are observed. Other driving factors potentially contribute to changes in HFMD dynamic transmission. These findings underscore the importance of implementing targeted policies aimed at early intervention, particularly when HFMD transmissibility begins to reach its peak.

## Author summary

Hand, foot, and mouth disease (HFMD) has become a global public health concern and demonstrates a strong capability for population-wide transmission. Compared to incidence, transmissibility can more directly indicate the effect of driving factors on dynamic transmission of infectious diseases and the critical period for prevention and control. In this study, we defined the HFMD epidemics, estimated transmissibility quantified as the instantaneous reproduction number ($R_{t}$), and employed a general additive model to investigate the association between potential factors and HFMD transmissibility in Zhejiang Province during 2013-2019. Furthermore, the case time series design was applied to investigate the association between temperature and HFMD incidence at township-level. Our results identified an inverted V-shaped relationship between temperature and HFMD incidence, as well as a similar nonlinear effect of temperature on HFMD transmissibility. Interestingly, the temperature corresponding to the highest transmissibility (around 19.69°C) was lower than the temperature where incidence risk peaked (around 28°C). Other factors including the depletion of susceptible individuals, school holidays, vaccination program, relative humidity and Normalized Difference Vegetation Index potentially contribute to changes in HFMD dynamic transmission. These findings could offer important insights into the implementation of targeted policies aimed at HFMD early intervention.

## 1. Introduction

Hand, foot, and mouth disease (HFMD) is a widespread infectious disease primarily affecting children under 5 years old, characterized by fever, rash or blisters on the hands and feet, and mouth ulcers [1]. While typically mild, severe cases can involve neurological or cardiorespiratory complications that could be fatal. Over the last decades, significant epidemics of HFMD have been observed in many countries in the Asian-Pacific region, including Vietnam, Singapore, Japan, and China [2]. HFMD is transmitted primarily through direct contact with infected individuals or contaminated objects, demonstrating a strong capability for population-wide transmission [3]. Current studies have estimated that the basic reproduction number ($R_{0}$) of HFMD ranges from 2.42 to 5.94 for different serotypes [4,5].

The epidemic of HFMD exhibits obvious seasonality, with peaks during the summer months in temperate Asia, while a biannual peak may occur during the spring and autumn in subtropical Asia [6]. This seasonal pattern underscores the potential impact of both meteorological factors (e.g., temperature, relative humidity, and precipitation) and cyclical human behaviors (e.g., school or kindergarten schedules) on HFMD incidence [7–10]. Temperature, in particular, may play an important role in the dynamic transmission of HFMD by influencing the environmental stability of enteroviruses, such as their survival and infectivity under specific conditions, and altering human interactions, such as the frequency of outdoor activities [11,12]. Supporting this, a time series study in East China reported an approximately M-shaped non-linear relationship between temperature and HFMD incidence [13]. Another meta-analysis that included 11 studies found that per 1°C increase in temperature was associated with a 5% increase in HFMD incidence [14].

However, current studies have mainly focused on the association between temperature and HFMD incidence, with no study having adopted transmissibility as the outcome variable, which could more directly indicate the effect of driving factors on HFMD dynamic transmission and the critical period for controlling HFMD [15]. The instantaneous reproduction number (R_*t*_) has been considered a reasonable proxy for summarizing the transmissibility of infectious diseases and can detect small changes in epidemic dynamics in nearly real-time, whereas such changes might not be directly reflected in the incidence data alone [16]. For example, a COVID-19 study by Pan A et al. demonstrated that R_*t*_ dropped significantly after public health interventions, preceding a decline in incidence [17]. Additionally, studies based on HFMD incidence estimated their exposure measurements by simply averaging each exposure variable across large regions (provinces or cities), rather than using values assigned to small areas (towns or villages). This may have resulted in improper exposure classification and lower estimation accuracy [18,19].

To address these knowledge gaps, this study collected high-quality surveillance data from Zhejiang Province, defined the HFMD epidemics, and estimated transmissibility quantified as $R_{t}$. Moreover, we used a novel methodology, the case time series (CTS) analysis, to assess the association between temperature and HFMD incidence at small-scale spatial patterns, and further employed a general additive model (GAM) to investigate the nonlinear effect of temperature on HFMD transmissibility.

## 2. Methods

### 2.1 Study area

**Fig 1:**
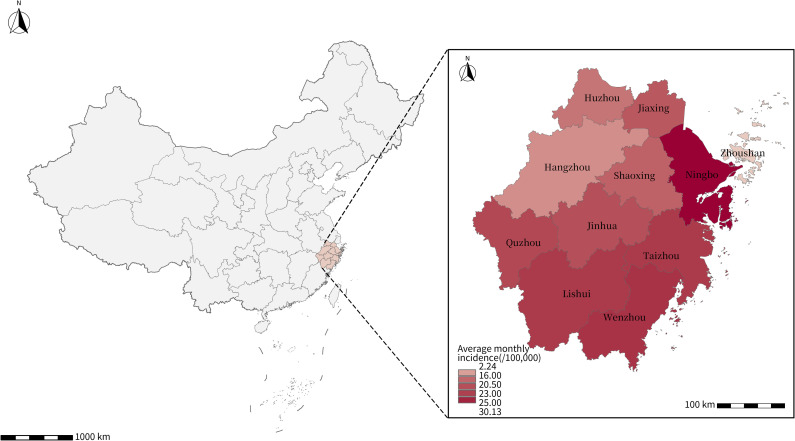
The geographic location of Zhejiang in China and the spatial distribution of HFMD average monthly incidence of 11 cities in Zhejiang from 2013 to 2019. The base layer of the map is from the National Catalogue Service for Geographic Information of the Ministry of Natural Resources of the People’s Republic of China (www.webmap.cn).

Zhejiang Province is situated on China’s southeastern coastline (118°E-123°E, 27°N-32°N), with humid air, temperate climate and thriving economy (Fig 1). Covering an area of 101,800 square kilometers and encompassing 11 cities (including 4 coastal cities, 6 non-coastal cities, and one archipelago), Zhejiang is known for its high population density within China. HFMD was one of the most common notifiable diseases among children in Zhejiang, with the average annual incidence of 1,430/100,000 between 2008 and 2017. Among the reported cases, 89.34% were aged 0-4 years old [20].

### 2.2 Daily series of HFMD incidence and meteorological measures

HFMD cases were diagnosed in accordance with China’s national diagnostic standards in hospitals, including both probable and laboratory-confirmed cases, and must be reported by healthcare professionals through the National Notifiable Infectious Diseases Reporting Information System (NNIDRIS) within 24 hours [21]. The surveillance data concerning HFMD incidence in Zhejiang Province from 2013 to 2019 was compiled from NNIDRIS, including information such as gender, age, occupation, residential location, and the date of onset. All data employed in this study were de-identified and anonymized prior to analysis, and no individual-level analysis was conducted. Ethical approval from institutional review boards was not required.

The hourly surface temperature (°C), dewpoint temperature (°C), total precipitation (m), and surface pressure (Pa) in Zhejiang Province with spatial resolution of 0.1°×0.1° were collected from the fifth generation of European Re-Analysis (ERA-5) gridded data and the daily average measures of the extracted grids were calculated. We used the Clausius-Clapeyron relation to calculate partial water vapor pressure and saturation vapor pressure based on temperature and dewpoint temperature, and derived relative humidity (%) as well as absolute humidity (g/m^3^) by their definitions [22]. Temperature and humidity are recognized as the two key factors of HFMD dynamic transmission, directly affecting both virus survival and human behavior.

The individual daily meteorological exposure of each HFMD case was estimated through the bilinear interpolation approach according to the longitude and latitude coordinates of their specific residential locations. Township-level measures were calculated as the arithmetic mean of daily measures of all cases on each day in each township, while the calculations of city-level and province-level measures were analogous. Notably, for a small area with no cases on a particular day, we averaged the values of all the grids contained in that township’s boundaries to estimate the exposure of meteorological factors for that day [23]. Additionally, we defined the cold season (November to March, including winter plus one month before and after it) and the warm season (May to September, including summer plus one month before and after it) based on the monthly average temperature distribution.

### 2.3 Covariate data sources

Considering that HFMD is one of the leading infectious diseases threatening children, it is essential to incorporate the school or kindergarten activity cycles of children. The school holidays included national holidays in China, covering New Year’s Day, Spring Festival, Labor Day, Dragon-Boat Festival, Mid-Autumn Festival, and National Day, along with winter and summer vacations for primary and secondary school students according to the school calendar issued by the local Education Bureau. Given that multiple previous studies suggest the potential association between green space and HFMD, we also considered Normalized Difference Vegetation Index (NDVI), a satellite-derived measure of vegetation greenness [24,25]. The monthly NDVI was obtained from NASA’s Moderate Resolution Imaging Spectroradiometer (MODIS) database with spatial resolution of 0.1°×0.1°.

### 2.4 HFMD epidemics and instantaneous reproduction number

Considering the dynamic changes in population susceptibility and variations in dominant enterovirus types, both of which could influence the accuracy of HFMD transmissibility estimates and the effectiveness of assessing potential driving factors’ impact, we first defined distinct HFMD epidemics during the study period instead of analyzing a single continuous time series. After employing spline smoothing on weekly reported incidences, we calculated the weekly growth rate by taking the derivative of the smoothed trajectory. Similar to methodologies in previous literature [26], a continuous increase (growth rate greater than 0) lasting at least five weeks was regarded as the beginning of the epidemic, and a continuous decrease (growth rate less than 0) lasting at least eight weeks before the next increase was regarded as the end. Ultimately, a total of 13 distinct epidemics were defined from 2013 to 2019. Furthermore, we gauged the epidemic size quantified as the total number of cases within the duration, as well as the timing and magnitude of the epidemic’s peak.

The instantaneous reproduction number R_*t*_is the expected number of secondary cases generated by an infected primary case, with R_*t*_>1 indicating epidemic expansion and sustained transmission, while R_*t*_<1 suggests declining transmission and eventual epidemic control. In published literatures, R_*t*_has been utilized as an outcome to assess the effect of different factors relating to infectious diseases, control measures, meteorological conditions and depletion of susceptible individuals during the epidemic, for instance [26–29]. The definition of serial interval is the timing between symptoms onset in a primary case and symptoms onset in his/her secondary cases. Given a distribution of the serial interval, we can estimate the daily reproduction numberR_*t*_for each epidemic within a Bayesian framework. Technical details of the calculation process of instantaneous reproduction number are provided in the S1 Text. To be specific, we assumed that the serial interval of HFMD follows a shifted Gamma distribution with a minimum value (shift) of 1 day, a mean of 3.7 days, and a standard deviation of 2.6 days [30]. Additionally, the smoothing time window for the estimation of R_*t*_was fixed at 14 days [31]. The above calculation process and subsequent statistical analysis of the relationship between transmissibility and driving factors were restricted to the periods of 13 identified HFMD epidemics.

### 2.5 Statistical analysis

Two parts of analysis were employed in our study. Firstly, we utilized the novel CTS design and distributed lag non-linear model (DLNM) to analyze the association between temperature and HFMD incidence using high-resolution data of small-scale patterns (i.e., townships) across the whole geographical domain. Furthermore, based on the calculation of $R_{t}$, we conducted a GAM to estimate the association between potential factors and HFMD transmissibility. Separate models were performed for each city, while seasonal (cold and warm season) and spatial (coastal and non-coastal cities) stratified analyses were also conducted.

Firstly, we analyzed the association between meteorological factors and HFMD incidence by conducting a CTS design, a novel methodology that allows the linkage and analysis of high-resolution data and the identification of small-scale risks across the whole geographical domain [19,32]. Specifically, this study treated each small-area (township) as the unit of analysis, calculated daily HFMD cases in each township, assessed daily exposure based on gridded exposure datasets as described in section 2.2, and then constructed time series data for each township.

Based on the above CTS design and township-specific temperature-incidence series data (2013-2019), we employed conditional quasi-Poisson regression and DLNM model to analyze the temperature-incidence associations at a higher-resolution level. We fitted the exposure-response function using a natural cubic spline with three internal knots placed at 10^th^, 75^th^, and 90^th^ percentiles of township-specific temperature distribution [33]. Considering the incubation period of infectious diseases, it is highly likely that the observed incidence risk could be affected by temperature exposure over a few days ago. Therefore, we defined the lag window as 0-21 days to fully capture the potential lagged effects of temperature [34], and used a natural cubic spline with three internal knots equally placed on the log scale. Additionally, we set the median of township-specific temperature (18.80°C) as the reference. Notably, the combined time series data were stratified by “township-year-month” to adjust for the varying baseline risks and trends across different spatiotemporal strata. During the model estimation process, strata with no recorded outcomes were excluded to reduce the risk of biased coefficient estimates and overdispersion parameters in the quasi-Poisson regression [19,32].

Some important confounders were controlled, including the natural splines of time with 7 degrees of freedom (df) per year as well as indicators of day of the week and school holiday to adjust long-term, seasonal and weekly trends. We also applied a natural spline for daily mean relative humidity with 3 df and incorporated an indicator term for the vaccination program officially launched at the end of 2016 in Zhejiang Province to control potential confounding effects. Finally, we reduced the bi-dimensional temperature-lag response curve to the one-dimensional cumulative exposure-response association at different lag windows to assess the effect of temperature on daily HFMD incidence. Algebraically, the expected counts of cases $E\left({\text{Y}}_{i,j,t}\right)$ at day *t* for each township *j* within city *i* were modelled separately as:


$$\begin{array}{c} ln\left[E\left({\text{Y}}_{i,j,t}\right)\right]\text{=}\alpha_{i}\text{+ cb}\left({\text{Temp}}_{i,j,t},lag,df_{1}\right)\text{+}ns\left(RH_{i,j,t},df_{2}\right)\text{+}V_{t}\text{+}W_{t}\text{+}P_{t}\\ \text{+}ns\left(Time_{t},df_{3}\right)\text{+}\varepsilon_{i,j,t} \end{array}$$

Additionally, the time varying instantaneous reproduction number ${\text{R}}_{t}$ was assumed to be proportional to $R_{0}$ and influenced by intrinsic drivers, such as the depletion of susceptible individuals, along with the extrinsic drivers, such as temperature. After taking the logarithm of both sides, we used GAM to investigate further the underlying association between the HFMD transmissibility and different driving factors. The final improved model incorporated long-term trends, seasonality, calendar effects, a smooth term of temperature, and other driving factors were built as follows:


$$\begin{array}{c} ln\left[{\text{R}}_{i,t}\right]\text{=}\ln\left(R_{i,0}S_{i,0}\right)\text{+}C_{i,t}\text{+ s}\left({\text{Temp}}_{i,t}\text{,}df_{1}\right)\text{+}ln\left(RH_{i,t}\right)\text{+}ln\left(NDVI_{i,t}\right)\\ \text{+}V_{t}\text{+}W_{t}\text{+}P_{t}\text{+ s}\left(Time_{t},df_{2}\right)\text{+}\varepsilon_{i,t} \end{array}$$

where ${\text{R}}_{i,t}$ means the instantaneous reproduction number of city *i* at day *t*, $C_{i,t}$ denotes the depletion of susceptible individuals namely the daily cumulative incidence of HFMD up to day *t*-1 at city *i*. ${\text{Temp}}_{i,t}$, $RH_{i,t}$, and $NDVI_{i,t}$ are respectively temperature, relative humidity, and normalized difference vegetation index of city *i* at day t, $V_{t}$ is the binary indicator for school holidays, $W_{t}$ is the dummy variable for the day of the week, $P_{t}$ is an indicator term for the vaccination program officially launched at the end of 2016 in Zhejiang Province, and $\varepsilon_{i,t}\sim N\left(0,\sigma^{2}\right)$ is the error term. The *df* for the smooth terms of temperature and time were set to 3 and 10 respectively, based on the approach of minimizing the sum of the absolute values of the partial autocorrelation function (PACF) of residuals, as done in previous studies [35,36]. In our analysis, $\ln\left(R_{i,0}S_{i,0}\right)$ might not be of immediate interest and can be viewed as the intercept. Other meteorological factors such as air pressure and precipitation were likewise tested but excluded from our final models to maintain stability and clarity.

### 2.6 Sensitivity analysis

Several sensitivity analyses were conducted to examine the robustness of our main results. Firstly, for CTS analysis, couples of specific knots of natural cubic spline for temperature were tested, and the *df* of natural splines of relative humidity and time were set to 4-6 and 6-9 respectively. Secondly, for R_*t*_ calculation, the end of an epidemic was defined as the weekly cases continuously decreasing for not less than 6-9 weeks before the next increase begins, and the mean of serial interval to calculate was assumed to be 2 days and 5 days respectively. Thirdly, for township-level meteorological factors, we used the average of all grid cells in a township regardless of case presence to verify the rationale of the exposure assessment method. Finally, other representations of humidity factors such as absolute humidity and a binary variable indicating the rainy season were both considered in GAM.

All statistical analyses were conducted in R version 4.2.2 (R Foundation for Statistical Computing, Vienna, Austria). The “EpiEstim” package was used to calculate the instantaneous reproduction number, while the “dlnm” and “mgcv” package were used to build our models.

## 3. Results

### 3.1 Descriptive results

**Fig 2:**
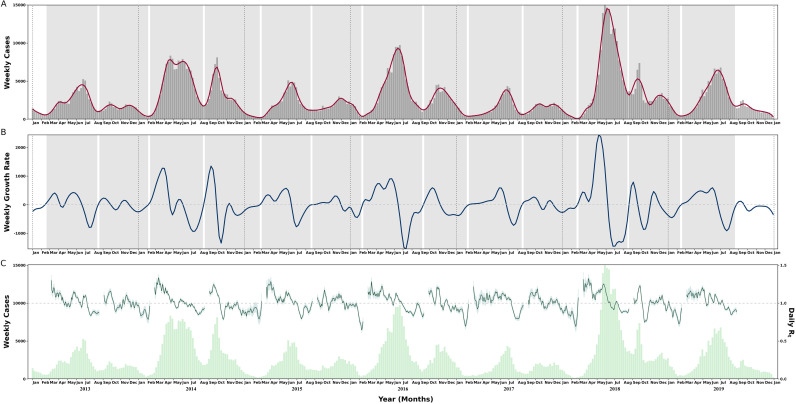
Identification of the HFMD epidemics and estimated transmissibility in Zhejiang from 2013 to 2019. **(A)** The number of HFMD cases by week and the smoothing spline curve. (B) The growth rate of the estimated daily number of HFMD from the spline curve. The grey blocks represent the main HFMD epidemic period identified while the black dotted lines represent breaks between different years. (C) The light green bars represent the number of HFMD cases by week and the dark green lines and the shade represent daily instantaneous reproduction number and its 95% CI in 13 epidemics. The dashed line represents the *R_t_* threshold of 1.

This analysis included 1,006,549 HFMD cases in Zhejiang during the study period. The distribution of these cases exhibited a seasonal and biannual pattern, and 13 epidemics were confirmed from 2013-2019 according to the definition in methods (Fig 2A and 2B). The monthly mean temperatures in Zhejiang hover around approximately 17.8°C (range 5.5 to 29.4), with city-specific averages in mainland ranging from 16.9°C (5.1 to 27.7) in Lishui to 18.0°C (7.3 to 28.1) in Wenzhou, reflecting a notably temperate climate. However, in Zhoushan, an archipelago surrounded by the sea, the mean monthly value is 21.8°C (7.7 to 29.2). Cities with a more significant disease burden of HFMD are primarily located in coastal areas with high population density. Ningbo, Wenzhou, and Taizhou ranked as the leading cities in the HFMD average monthly incidence, while the incidence in Zhoushan city remained low during the study period (Table 1). From epidemic curves, we observed that the HFMD epidemics in 2014, 2016 and 2018 were characterized by a higher cumulative number of cases, earlier peak timing, and larger peak magnitude compared to other years. During each year, the rapid increase in HFMD cases generally initiated at spring around March, and the predominant peaks were usually in the summer months, spanning from May to July. Additionally, relatively mild epidemic was frequently occurred from around late August and extended into the winter months. We calculated daily R_*t*_ restricted to 13 HFMD epidemics and described the begin timing, size, peak timing, peak magnitude and daily temperature distribution in each HFMD epidemic (Fig 2C and Table 2). The estimated R_*t*_ in spring-summer epidemics was significantly higher than that in autumn-winter epidemics.

**Table 1:** Descriptive statistics by regions for HFMD cases information (monthly cases, monthly incidence, and distributions of gender, age, and care status), and monthly temperature distributions in Zhejiang from 2013 to 2019.

Region	Total population	HFMD cases information							Monthly temperature (°C)					
		Monthly cases (N)	Monthly incidence (/100,000)	Male (%)	0-2 years old (%)	3-5 years old (%)	Scattered children (%)	Nursery children (%)	Mean ± SD	Min	P_25_	Median	P_75_	Max
Total	55,722,856	11,984	21.51	58.66	58.31	29.11	66.43	29.18	17.8 (±7.2)	5.5	11.3	18.0	24.2	29.4
Hangzhou^**b**^	9,128,130	1,093	11.97	58.54	60.03	26.43	68.27	25.90	17.2 (±8.2)	3.6	10.2	18.0	24.1	31.5
Huzhou^**b**^	2,953,350	474	16.05	57.71	52.65	30.80	59.44	34.15	17.1 (±8.1)	2.7	11.1	17.8	24.0	31.1
Jiaxing^**c**^	4,613,429	950	20.59	58.53	50.88	33.03	57.92	36.00	17.3 (±8.1)	3.8	10.3	18.0	24.1	31.3
Jinhua^**b**^	5,486,096	1,201	21.89	59.92	66.28	25.48	71.33	25.91	17.5 (±8.0)	4.7	10.3	18.2	24.5	30.6
Lishui^**b**^	2,151,459	536	24.91	57.83	65.32	25.29	73.62	23.31	16.9 (±7.2)	5.1	10.6	17.4	23.5	27.7
Ningbo^**c**^	7,840,059	2,362	30.13	59.11	55.92	31.24	61.45	34.07	17.4 (±7.7)	5.1	10.2	18.0	24.2	30.1
Quzhou^**b**^	2,151,472	495	23.01	57.30	63.49	28.29	66.48	31.14	17.8 (±7.8)	5.4	11.7	18.4	24.7	30.8
Shaoxing^**b**^	4,984,770	948	19.02	58.59	53.28	31.87	65.06	29.87	17.3 (±8.2)	4.0	10.0	18.2	24.3	31.3
Taizhou^**c**^	6,071,604	1,516	24.97	59.82	59.92	28.14	77.72	18.88	17.7 (±7.3)	5.9	10.8	18.1	24.5	29.0
Wenzhou^**c**^	9,181,470	2,383	25.95	60.55	59.14	28.51	70.33	26.02	18.0 (±6.8)	7.3	11.3	18.2	24.3	28.1
Zhoushan^**a**^	1,161,017	26	2.24	57.40	54.46	31.17	59.12	35.74	21.8 (±5.2)	7.7	19.9	22.8	25.7	29.2

Note: a: archipelago; b: non-coastal cities; c: coastal cities; Scattered children: children not attending any formal childcare institutions (e.g., nurseries, kindergartens); Nursery children: children attending formal childcare institutions; SD, standard deviation.

**Table 2:** Descriptive statistics by defined epidemics for estimated *R_t_* (and 95% CI), begin timing, size, peak timing, peak magnitude and daily temperature distributions in Zhejiang from 2013 to 2019.

Epidemic No.	Mean estimated *R_t_* (95% CI)	Begin timing, (yyyy-mm)	Size, no. of cases	Peak timing, (yyyy-mm)	Peak magnitude, no. of cases	Daily temperature (°C)					
						Mean ± SD	Min	P_25_	Median	P_75_	Max
1	1.024 (0.994,1.055)	2013-02	65,165	2013-06	5,274	21.0 (±7.4)	4.8	15.2	21.7	27.9	32.8
2	0.980 (0.943,1.019)	2013-08	34,263	2013-09	2,323	15.5 (±7.7)	1.7	9.2	14.7	22.2	29.2
3	1.036 (1.013,1.060)	2014-02	133,296	2014-04	8,349	19.5 (±6.9)	1.2	15.1	21.2	25.1	29.1
4	0.962 (0.927,0.998)	2014-08	70,040	2014-09	8,129	15.1 (±6.9)	2.8	8.5	14.6	20.8	27.8
5	1.037 (1.003,1.072)	2015-03	57,884	2015-06	5,109	21.5 (±5.6)	6.7	18.7	22.5	25.9	29.9
6	0.987 (0.954,1.021)	2015-08	42,354	2015-11	2,990	14.9 (±7.0)	-3.2	9.4	15.7	21.3	26.2
7	1.025 (0.997,1.053)	2016-02	121,570	2016-06	9,739	21.5 (±6.7)	4.9	16.7	22.6	27.4	31.2
8	0.979 (0.949,1.009)	2016-09	51,846	2016-10	4,564	14.3 (±6.5)	3.6	8.7	13.2	20.8	26.2
9	1.024 (0.982,1.067)	2017-02	42,328	2017-06	4,348	20.7 (±6.9)	6.3	14.9	21.3	27.4	31.7
10	0.977 (0.935,1.020)	2017-08	34,004	2017-12	2,173	14.0 (±8.2)	-1.0	7.7	12.5	21.5	29.6
11	1.073 (1.045,1.101)	2018-02	162,860	2018-05	15,023	21.9 (±6.1)	7.0	17.6	22.8	27.5	30.5
12	0.964 (0.936,0.992)	2018-08	73,190	2018-09	7,386	14.5 (±7.8)	1.4	7.3	13.9	20.3	28.4
13	1.038 (1.007,1.069)	2019-02	86,675	2019-07	6,797	20.0 (±6.8)	5.3	14.4	21.4	25.7	30.4

Note: SD, standard deviation; CI, confidence interval.

### 3.2 The association between temperature and HFMD incidence

**Fig 3:**
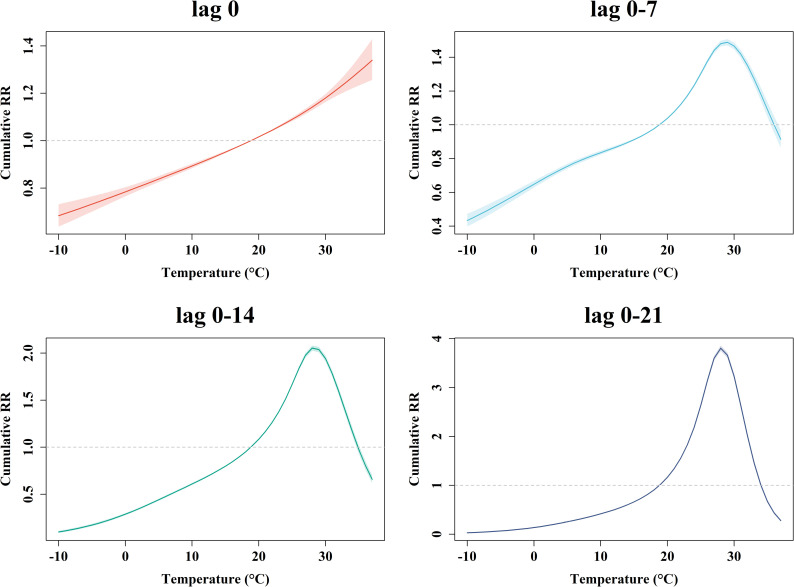
The cumulative exposure-response relationships between temperature and HFMD incidence at different lag windows in CTS analysis. The solid lines and shaded areas indicate lag-specific relative risk for temperature and its 95%CI.

Based on the CTS analysis and high-resolution measurements assigned at small-area level, we investigated the effect of temperature on HFMD incidence in both lag and exposure dimensions. The cumulative exposure-response curves presented relatively consistent characteristics (Fig 3). Specifically, at initial time (lag 0), the risk rose steeply with increasing temperature, showing a clear upward trend. Other lag-specific curves showed a pattern of rising followed by a decline, peaking around 28-29°C. The overall (lag 0-21 days) cumulative exposure-response curve illustrated that risk effects are observed between 18.81-34.03°C, with the highest cumulative relative risk (RR) of 3.81 (3.75, 3.86) at 28°C, underscoring the intensifying impact of prolonged high temperatures on HFMD risk. Additionally, the magnitude of the risk effect at the peak compared to the reference temperature increased over time, from a cumulative RR of 1.49 (95%CI: 1.47-1.51) at 7-day lag to 2.05 (2.03-2.08) at 14-day lag. To illustrate detailed discrepancies across cities, we also compared the city-specific overall (lag 0-21) cumulative exposure-response curves in S1 Fig. Most curves showed an inverted V-shape, consistent with the curve of the total province, with the highest cumulative risk occurring around 28-29°C. Additionally, each of the 21 lag-response associations was reported in S1 and S2 Tables.

### 3.3 The association between temperature and HFMD transmissibility

**Fig 4:**
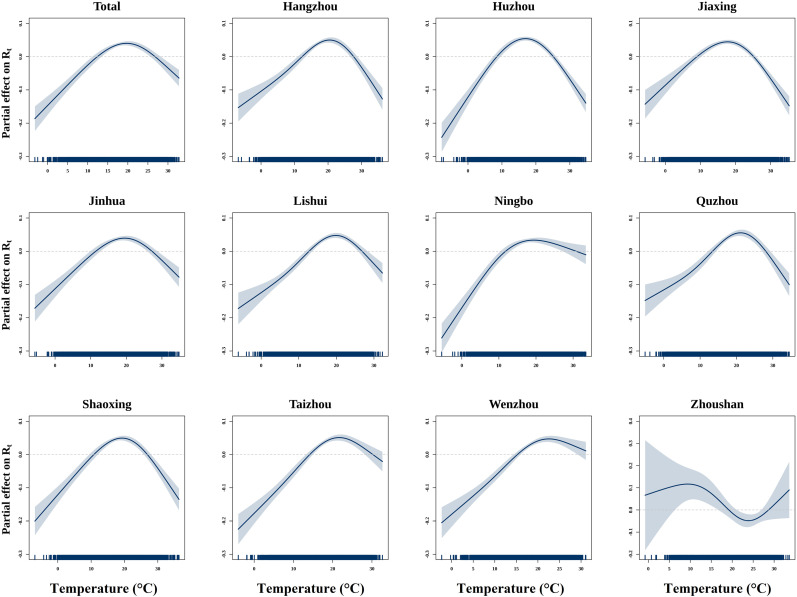
Partial effects of temperature on HFMD transmissibility in 11 cities in Zhejiang using GAMs. The solid lines indicate region-specific temperature-*R_t_* curves. The 95%CI are reported as shaded areas.

We discovered an inverted V-shaped correlation between ambient temperature and HFMD transmissibility in Zhejiang and separately investigated the effects of temperature for 11 cities. Specifically, a positive correlation was evident within the temperature range of 12.43°C to 26.77°C, where the highest transmissibility occurred at 19.69°C. Furthermore, among the 11 cities analyzed, 10 cities displayed a similar inverted V-shaped patter, with temperatures associated with the highest risk consistently around 19.92°C. The lowest temperature was observed in Huzhou at 17.12°C, while the highest was in Wenzhou at 22.67°C. However, the curve of Zhoushan demonstrated a trend in which both low and high temperatures elevated the risk (Fig 4). Notably, Zhoushan’s unique geographic isolation leads to highly sporadic HFMD cases, which may reduce the precision of R_*t*_ estimation compared to mainland cities, and the observed temperature-transmissibility association may be not accurate.

### 3.4 Effects of temperature on HFMD stratified by regions and seasons

**Fig 5:**
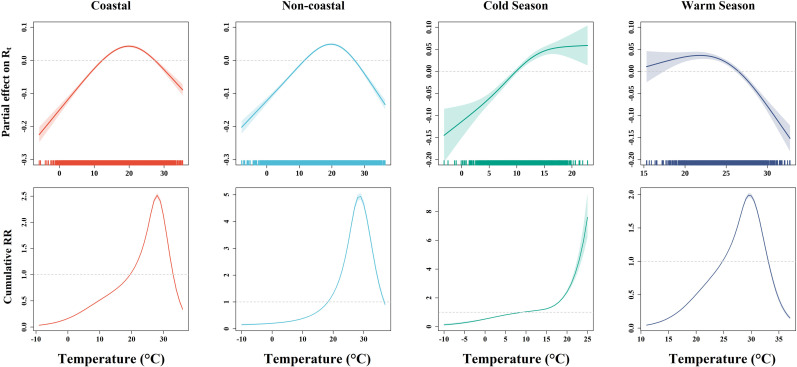
The exposure-response relationships between temperature and HFMD transmissibility as well as incidence stratified by regions and seasons. The solid lines indicate partial effect on *R_t_* or overall cumulative relative risk for temperature. The 95%CI are reported as shaded areas.

Similar to the main result, inverted V-shaped associations between temperature and HFMD transmissibility were observed in both coastal and non-coastal cities, with the highest R_*t*_ occurring at 19.97°C and 19.88°C respectively. Additionally, the overall (lag 0-21) cumulative exposure-response curves between HFMD incidence and temperature in both coastal and non-coastal cities exhibited a similar pattern, characterized by a continuous increase followed by a decline, peaking at 28°C and 29°C respectively. However, the highest risk of HFMD compared to the reference temperature for non-coastal cities (cumulative RR=4.94, 95% CI: 4.81, 5.06) was higher than that of coastal cities (cumulative RR=2.52, 95% CI: 2.47, 2.56) (Fig 5).

For the seasonal stratified analysis, we observed a continuous increasing between R_*t*_ and temperature in cold season, and a gradually decrease trend from around 21.84°C in warm season. As for temperature-incidence association, we observed a steep increase in overall (lag 0-21) cumulative HFMD risk with temperature in the cold season around 20-25°C, while an inverted V-shape relationship peaking at 29.5°C (cumulative RR=1.99, 95% CI: 1.96, 2.02) was noted during the warm season. Notably, the magnitude of effects was higher in the cold season than the warm (Fig 5).

### 3.5 Other driving factors of HFMD transmissibility

The analysis of GAM also suggested that other potential factors played important roles in the transmission of HFMD. To be specific, each 10^5^ cases increase in the depletion of susceptible individuals resulted in a 4.84% (95% CI: 3.92%, 5.76%) reduction in HFMD transmissibility and the school holiday was associated with a 9.94% (95% CI: 8.95%, 10.91%) reduction in the instantaneous reproduction number. The implement of vaccination program after 2017 reduced 8.75% (95% CI: 4.99%, 12.35%) of HFMD transmissibility. We also found a significant negative association between relative humidity and R_*t*_ (-0.06, 95% CI: -0.09, -0.03), as well as a significant positive association between NDVI and HFMD transmissibility (0.08, 95% CI: 0.01, 0.16) (Table 3). Furthermore, the direction of these factors’ effect on R_*t*_ in models stratified by different cities basically identical (S3 Table). We did not find significant correlations between other meteorological factors and the instantaneous reproduction number of HFMD.

**Table 3:** Estimated variation in *R_t_* associated with other driving factors.

Covaries	Variation in *R_t_* (%)	95% CI
**Each unit increase**		
Depletion of susceptible, 10^5^	-4.84	(-5.76, -3.92)
School holiday		
No	Ref	
Yes	-9.94	(-10.91, -8.95)
Weekday		
No	Ref	
Yes	-0.04	(-0.92, 0.84)
Vaccination program		
No	Ref	
Yes	-8.75	(-12.35, -4.99)
**1% per increase**		
Relative humidity	-0.06	(-0.09, -0.03)
NDVI	0.08	(0.01, 0.16)

Note: CI, confidence interval; NDVI, normalized difference vegetation index.

### 3.6 Sensitivity analysis

The results of sensitivity analysis demonstrated that when changing the knots of natural cubic spline of temperature or extending the dfs for relative humidity and time between 4-6 and 6-9 respectively, the overall effect of temperature was stable in CTS analysis (S5, S6 and S7 Tables). When changing the mean of serial intervals (2 or 5 days) and the definition of epidemic ending (decrease not less than 6-9 weeks respectively), the curves reflecting the effect of temperature on HFMD transmissibility were similar to those from the principal analysis (S8 Table). Moreover, when using conventional exposure assessment method, temperature effect estimates were similar to and slightly higher than original estimates (S4 Table). Results from models introduced other representations of humidity showed similarity with our main findings (S9 Table).

## 4. Discussion

This is the first study to estimate the effect of temperature on HFMD transmissibility, which could better identify the optimal conditions for HFMD dynamic transmission and the critical period for HFMD early intervention. We observed an inverted V-shaped association between temperature and HFMD incidence with the highest overall (lag 0-21) cumulative RR at 28°C. Additionally, we discovered a similar nonlinear association between temperature and HFMD transmissibility with the highest R_*t*_ occurring at 19.69°C. Notably, we found the temperature corresponding to the highest transmissibility was lower than the temperature where incidence reached the peak. Moreover, the depletion of susceptible individuals, school holidays, vaccination program, relative humidity and NDVI could contribute to changes in transmissibility of HFMD during an epidemic.

Our results in CTS analysis demonstrated an inverted V-shaped association between temperature and HFMD incidence with the highest cumulative RR of 3.81 at 28°C, which was similar to previous studies. One multicity study from China reported a similar inverted V-shaped association between temperature and HFMD incidence, and further showed that the risk of HFMD had a gradual increase until it peaked at the 91th percentile of temperature with reference to the median (RR = 1.30, 95% CI: 1.23, 1.37) [12]. Another study of 17 cities in Shandong, China also observed the temperature effects on HFMD incidence and the result showed an approximately inverted V-shaped association with the highest RR of 1.40 (95% CI: 1.15, 1.69) at 23.2°C [37]. The stronger relationship observed in our study compared to previous studies could be attributed to regional heterogeneity including differences in host population susceptibility and other environmental characteristics, as well as temporal variability. While caution is needed in generalizing our findings to another location, they still provided reliable and valuable information for targeted HFMD prevention and control and early warning in the local context.

We further observed that temperature exhibited a similar inverted V-shaped association with HFMD transmissibility in GAM. Although no studies have examined this relationship, our results were supported by both laboratory evidence and population studies that adopted incidence as the outcome variable. The sensitivity of the HFMD pathogen to temperature has been confirmed, for example, a vitro study observed that the survivability of enteric viruses enhanced at 22°C compared to 30°C in artificial seawater [38]. In another animal study, exposure to temperatures 3°C above the optimal for Enterovirus 71 (EV71), a major pathogen causing HFMD and severe cases, resulted in milder neuropathological changes and restricted viral spread [39]. A cellular experiment evaluating the virulence variation during the temperature-adaptive evolution of EV71 discovered that the cell inhibition rates of cold-adapted and heat-adapted strains were both lower than those of the normothermic-adapted strain [40]. Moreover, the results in the previous part of our analysis and population studies conducted in other regions also reported analogous inverted V-shaped relationships between temperature and HFMD incidence [41,42].

Several mechanisms could explain the nonlinear temperature-HFMD association. Firstly, within a suitable range, higher temperature will promote the reproduction, persistence, and transmission of the virus [43]. However, the activation of enteroviruses may diminish as temperatures drop below the threshold level [13], and the extremely high temperature is largely considered as the major factor determining virus inactivation in the environment [44,45]. Secondly, the frequency of outdoor activities and social contact with other susceptible individuals of children tend to increase at more moderate temperatures, whereas during extremely hot days or in winter, people prefer to stay indoors [46,47].

Interestingly, the temperature corresponding to the highest transmissibility (around 19.69°C) was lower than the temperature where incidence risk peaked (around 28°C). Current methodology evidence could explain this discrepancy. Specifically, the incidence (absolute number of cases) is a cumulative outcome influenced by the incubation period, reporting lag, and individual susceptibility factors. In contrast, R_*t*_ is an ideal metric to reflect the direct and immediate changes in the pathogen’s ability to spread between individuals [48,49]. By combining the characteristics of these two indicators with our findings, it is reasonable to infer that, as the temperature approaches a suitable range, the transmissibility of HFMD pathogen is affected first. This increased transmissibility subsequently leads to a rise in observed incidence, reflecting a cumulative process that involves not only the direct effect of transmissibility but also the time required for infections to progress, the delays associated with case reporting, and the dynamic interactions within host populations. Between the optimal temperatures for transmissibility and incidence, human behaviors likely play a crucial role in amplifying incidence, even when viral survival or reproduction is reduced. For example, susceptible children are more likely to engage in outdoor activities and have increased social interactions within this temperature range, resulting in longer and more frequent exposures, which in turn contribute to higher observed incidence. Moreover, as cases accumulate, the depletion of the susceptible population and the subsequent development of immunity serve as natural barriers to further transmission. This leads to a gradual decline in transmissibility until the pool of susceptible individuals is replenished over time. This could also explain why the size and peak magnitude of HFMD epidemics in autumn are generally smaller than those observed in spring.

For the seasonal stratified results, the associations between temperature and cumulative HFMD risk were found to be stronger in the cold season compared to the warm season, which was similar to the previous evidence [50]. The seasonal differences may be attributed to the fact that during the cold season, when temperatures favor the viral survival, children engage in more indoor social activities, which enables the virus persist on surfaces for longer periods and demonstrated significant impact at longer lag days [51]. Moreover, a similar but stronger association between temperature and cumulative HFMD risk was observed in non-coastal cities compared to coastal cities. The similar pattern of temperature and transmissibility indicated that the virus survives for a similar duration at the same temperature, while the effect of temperature on HFMD risk was greater in non-coastal areas, which might be attributable to the relatively lower economic status and healthcare conditions in inland cities [52]. These findings highlight the importance of implementing region-specific health policies for targeted interventions and resource allocation.

Our results also demonstrated that the reduction of HFMD transmissibility was associated with the depletion of susceptible, school holidays, vaccination program, higher relative humidity and lower NDVI. The strong negative correlation between the depletion of susceptible and HFMD R_*t*_ is intuitive, since population immunity is one of the key drivers affecting the transmission [53]. Once an epidemic is underway, the proportion of susceptible individuals gradually decreases, meaning population immunity is increasing, thereby resulting in transmissibility decreasing until the end of the epidemic. School holidays were also associated with the reduction in transmissibility, because kindergarten children and students have higher contact rate during school hours [27]. Additionally, the protective effect observed from the vaccination program could indirectly support the effectiveness of the EV71 vaccine. We observed a weak negative correlation between relative humidity and R_*t*_. A possible explanation is that the rapid evaporation in low-humidity environment facilitates suspension of the viruses in droplets [42]. There are few studies on NDVI and HFMD risk. The positive correlation we observed may be due to children living or studying in greener areas being more physically active, leading to more frequent contact with other susceptible individuals [54].

Our study had several strengths. Firstly, our exposure assessment approach for environmental factors was based on gridded data and the individual latitude and longitude coordinates of cases, which can provide a more accurate estimation of individual-level exposure compared to using fixed-site data. Secondly, we applied the novel CTS design assigning exposure measurements to small areas rather than simply averaging their values across large regions, which made it possible to estimate the exposure-response function with high-resolution township-level data, hence reduced the exposure misclassification, increased the precision of the estimates, and reduced the risk of ecological fallacies [55]. Thirdly, this study was based on a large sample size and extended duration, allowing for a robust analysis.

Some limitations should be acknowledged. Firstly, under-reporting is inevitable in a surveillance system, since patients with mild symptoms may not seek formal medical treatment, which may lead to the underestimation of temperature effects. Secondly, while incorporating a simple binary indicator for the EV71 vaccination program, which officially launched at the end of 2016 in Zhejiang Province and had been verified as effective in reducing EV71 cases [56], we may have overlooked variations in vaccination coverage over time and across different regions, limiting our ability to fully capture its impact. Thirdly, etiological information was not taken into account in our analysis, and the transmissibility and temperature sensitivity among different serotypes of virus may be different, leading to potential biases in our results.

## 5. Conclusion

This study observes an inverted V-shaped relationship between temperature and HFMD incidence, as well as a similar nonlinear effect of temperature on HFMD transmissibility. By comparing the two inverted V-shaped curves, the threshold temperature window (between 19.69°C and 28°C) for HFMD early intervention is identified. Other significant driving factors of HFMD transmissibility include depletion of susceptible individuals, school holidays, vaccination program, relative humidity and NDVI. In conclusion, these findings offer important insights into the implementation of targeted policies to reduce the HFMD burden.

## Supporting Information

S1 Text The calculation of instantaneous reproduction number. (DOCX)

S2 Text Lag-response relationship between temperature and HFMD incidence. (DOCX)

S1 Fig The overall (lag 0-21) cumulative exposure-response relationships between temperature and HFMD incidence in 11 cities in CTS analysis.
(TIF)

S2 Fig The lag-response relationships between temperature and HFMD incidence of different temperatures (0°C, 4°C, 29°C, 31°C, i.e., 1th, 5th, 95th, and 99th percentile of daily mean temperature respectively) in CTS analysis.
(TIF)

S1 Table The effect of temperature (relative risk) on HFMD incidence for each lag in CTS analysis.
(XLSX)

S2 Table The effect of temperature (cumulative relative risk) on HFMD incidence for each lag window in CTS analysis.
(XLSX)

S3 Table Estimated variation in *R* *_t_* (%, and its 95% CI) associated with other environmental factors using GAMs stratified by different cities.
(XLSX)

S4 Table The effect of temperature (cumulative relative risk, and its 95% CI) on HFMD incidence when changing the exposure assessment method in CTS analysis.
(XLSX)

S5 Table The effect of temperature (cumulative relative risk, and its 95% CI) on HFMD incidence when changing the knots specification of natural spline for temperature in CTS analysis.
(XLSX)

S6 Table The effect of temperature (cumulative relative risk, and its 95% CI) on HFMD incidence when extending the degrees of freedom for relative humidity (RH) between 4-6 in CTS analysis.
(XLSX)

S7 Table The effect of temperature (cumulative relative risk, and its 95% CI) on HFMD incidence when extending the degrees of freedom per year for time (for seasonal control) between 6-9 in CTS analysis.
(XLSX)

S8 Table Estimated variation in *R* *_t_* (%, and its 95% CI) associated with other driving factors when the mean of serial interval was assumed 2 or 5 days and the end of an epidemic was defined as decreasing for not less than 6-, 7- or 9-weeks using GAMs.
(XLSX)

S9 Table Estimated variation in *R* *_t_* (%, and its 95% CI) associated with other driving factors when considering other representations of humidity factors using GAMs.
(XLSX)

S10 Table The effect of temperature (cumulative relative risk, and its 95% CI) on HFMD incidence when not omitting empty township-year-month strata (without observed cases) in CTS analysis.
(XLSX)
