# Supplementary material for: Effects of temperature and environmental covariates on the dynamic transmission of hand, foot, and mouth disease in Zhejiang, China
Source: PLoS Negl Trop Dis. 2025 Mar 18;19(3):e0012884. doi: 10.1371/journal.pntd.0012884 (PMC11918438; doi:10.1371/journal.pntd.0012884)
Supplement: S1 Text — (DOCX) [file pntd.0012884.s001.docx]

**Supplementary Material**

***The calculation of instantaneous reproduction number***

The basic reproduction number $R_{0}$ is the expected number of secondary cases that each infected primary case would infect in an entirely susceptible population without any intervention. The instantaneous reproduction number $\text{R}_{t}$, defined as the same measure if the conditions remained as they were at time t, is typically smaller than the value of $R_{0}$, and reflects the impact of potential factors rather than just the pathogen itself [1]. The relationship of $R_{0}$ and $\text{R}_{t}$ can be expressed as follows:

$$\begin{aligned} \text{R}_{t}=R_{0}S_{t}\#\left( 1 \right) \end{aligned}$$

where St denotes the proportion of susceptible population at time t.

As the key variable commonly used to characterize pathogen transmissibility during an epidemic and indicate whether the epidemic is under control ($\text{R}_{t}$<1), $\text{R}_{t}$ has been utilized as an outcome to assess the effect of different factors relating to infectious diseases, control measures, meteorological condition and depletion of susceptible individuals during the epidemic, for instance [2-4]. Accounting for these potential factors, the time-varying reproduction number $\text{R}_{t}$ can be:

$$\begin{aligned} &\text{R}_{t}\approx R_{0}S_{0}e^{\beta_{C}\text{*}C_{t}}\prod_{i} \beta_{1i}x_{1i}\prod_{j} e^{\beta_{2j}x_{2j}}\#(2) \end{aligned}$$

where $S_{0}$ denotes the initial proportion of susceptible population at the beginning of epidemic, $\{x_{1i}\}\left( i=1,2,\ldots\right)$ and $\{x_{2j}\}\left( j=1,2,\ldots\right)$ denote related factors whose association with transmissibility were better representation in exponential form and exponential form respectively. Only the linear association of factors are taken into consideration in this formula as a matter of convenience.

Considering the transmission events are independent, the number of secondary cases that a primary case infected can be regard as a statistic process obeying Poisson distribution. Denoting $n_{t}$ the incidences number at time t and $I\left( T \right)={\{n_{t}\}}_{0<t<T}$ the daily counts of patients whose symptoms appear before time T. The serial interval (timing between symptoms onset in a primary case and symptoms onset in his/her secondary cases) has a known probability distribution $w(.)$. A Bayesian statistical framework[5] described by Cauchemez et al. and the likelihood-based estimation procedure[6] proposed by Wallinga et al. are combined to infer the posterior distribution of Rt conditional on $I(T)$ and $w(.)$, and will estimate one value per time step (typically per day) of incidence. At each time step t, we calculate the average reproduction number $R_{t,\tau}$ over a time window of length τ ending at time t to make the resulting $R_{t}$ estimates to be less variable and more precise.

**References**

1. Cori, A., et al., *A new framework and software to estimate time-varying reproduction numbers during epidemics.* Am J Epidemiol, 2013. **178**(9): p. 1505-12.

2. te Beest, D.E., et al., *Driving factors of influenza transmission in the Netherlands.* American journal of epidemiology, 2013. **178**(9): p. 1469-1477.

3. Ali, S.T., et al., *Meteorological drivers of respiratory syncytial virus infections in Singapore.* Sci Rep, 2020. **10**(1): p. 20469.

4. Charnley, G.E.C., et al., *Cholera past and future in Nigeria: Are the Global Task Force on Cholera Control's 2030 targets achievable?* PLoS Negl Trop Dis, 2023. **17**(5): p. e0011312.

5. Cauchemez, S., et al., *Real-time estimates in early detection of SARS.* Emerging Infectious Diseases, 2006. **12**(1): p. 110-113.

6. Wallinga, J. and P. Teunis, *Different epidemic curves for severe acute respiratory syndrome reveal similar impacts of control measures.* Am J Epidemiol, 2004. **160**(6): p. 509-16.
