# Supplementary material for: Effects of temperature and environmental covariates on the dynamic transmission of hand, foot, and mouth disease in Zhejiang, China
Source: PLoS Negl Trop Dis. 2025 Mar 18;19(3):e0012884. doi: 10.1371/journal.pntd.0012884 (PMC11918438; doi:10.1371/journal.pntd.0012884)
Supplement: S2 Text — (DOCX) [file pntd.0012884.s002.docx]

**Supplementary Material**

***Lag-response relationship between temperature and HFMD incidence***

In our study, the temperature-incidence association persisted throughout the 21-day lag period. The relative risks (RRs) at both low temperatures (1st and 5th percentiles) and high temperatures (95th and 99th percentiles) exhibited significant immediate effects on HFMD incidence on the current day (lag 0) (S2 Fig). However, these effects were followed by opposite associations in the subsequent 1-3 days. The low temperatures effects fluctuated over shorter 7-9 days lag periods before stabilizing, whereas high-temperature effects exhibited prolonged fluctuations. This suggests that HFMD intervention may need to be sustained for longer durations in hot days.

Our results suggest that the temperature effect reached its maximum value on the current day (lag 0) but dropped sharply over the following 1-3 days, and the effect observed on lag 0 was approximately equal to the cumulative effect over lags 0-3. This pattern can be explained by a compensatory mechanism, for example, high temperatures may accelerate symptom onset in individuals who are already infected but not yet symptomatic [1]. Consequently, we believe that the effects observed at longer lags represent the “true” effect of temperature on HFMD incidence.

The unique lag-response relationship observed in our results (temperature effect is increasingly stronger over the lag period) may be attributed to discrepancy in study design. The case time series design provided a larger sample size and enabled more comprehensive effect capture. The standard time series analyses with aggregated data typically report associations over narrower temperature ranges, as extreme temperatures are averaged out, resulting in slightly lower risk estimates and failing to capture residual effects [2]. Additionally, regional differences in climate conditions and population characteristics may also play an important role. For instance, Zhejiang Province’s climate conditions might prolong the active period of the virus under high temperatures, and high population mobility could lead to cases being detected or reported after a longer lag. Similar phenomena have been observed in other studies on HFMD conducted in Zhejiang [3-5].

**References**

1. Yi, X., et al., *The temporal characteristics of the lag-response relationship and related key time points between ambient temperature and hand, foot and mouth disease: A multicity study from mainland China.* Sci Total Environ, 2020. **749**: p. 141679.

2. Gasparrini, A., *A tutorial on the case time series design for small-area analysis.* BMC Med Res Methodol, 2022. **22**(1): p. 129.

3. Zhang, R., et al., *Daily mean temperature and HFMD: risk assessment and attributable fraction identification in Ningbo China.* J Expo Sci Environ Epidemiol, 2021. **31**(4): p. 664-671.

4. Huang, R., et al., *Effects of Meteorological Parameters and PM10 on the Incidence of Hand, Foot, and Mouth Disease in Children in China.* Int J Environ Res Public Health, 2016. **13**(5).

5. Chen, Y., et al., *Seasonality and Meteorological Factors Associated With Different Hand, Foot, and Mouth Disease: Serotype-Specific Analysis From 2010 to 2018 in Zhejiang Province, China.* Front Microbiol, 2022. **13**: p. 901508.
