# Supplementary figures and images for: Effects of temperature and environmental covariates on the dynamic transmission of hand, foot, and mouth disease in Zhejiang, China
Source: PLoS Negl Trop Dis. 2025 Mar 18;19(3):e0012884. doi: 10.1371/journal.pntd.0012884 (PMC11918438; doi:10.1371/journal.pntd.0012884)

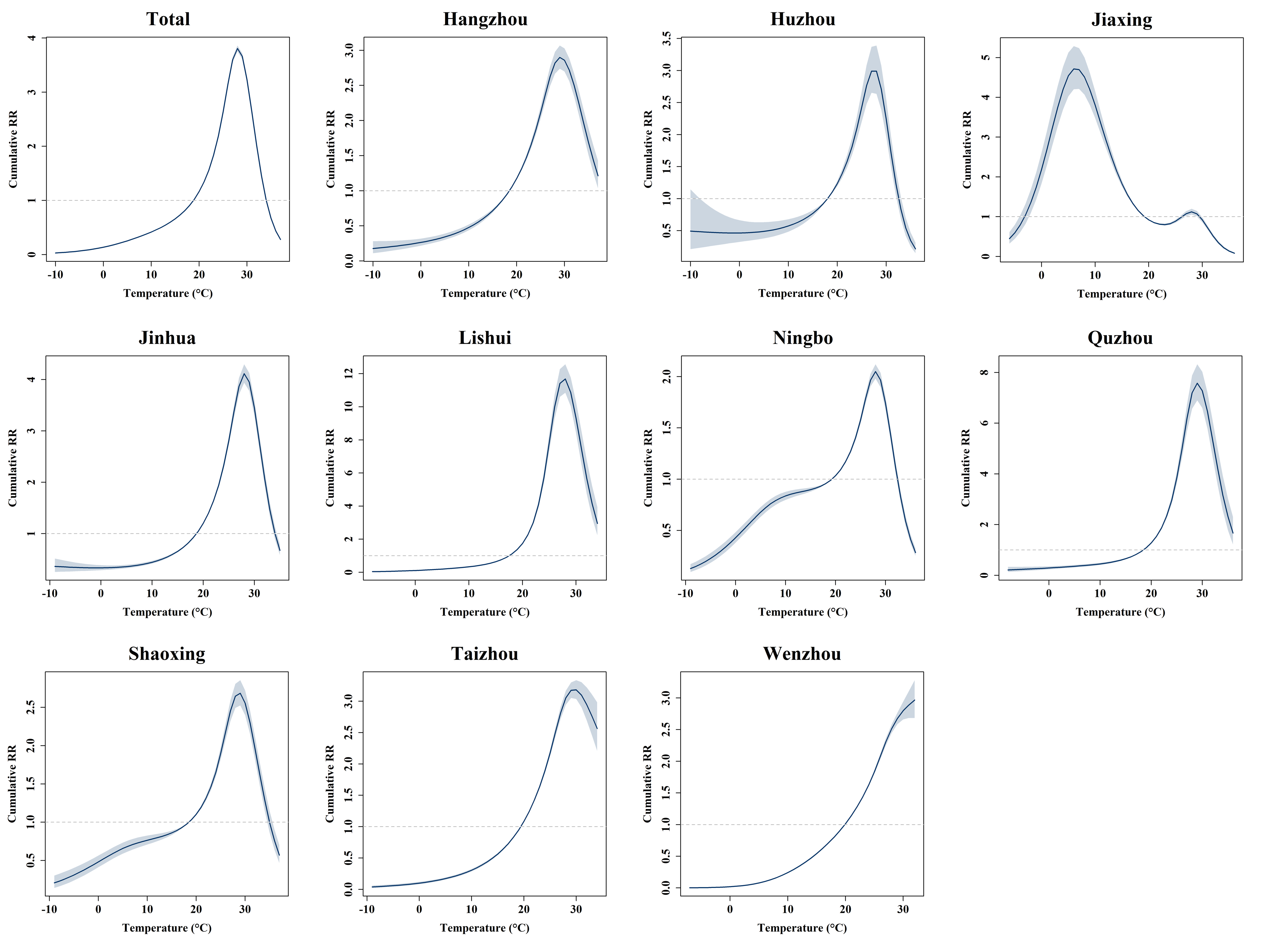

Supplement: S1 Fig — (TIF) [file pntd.0012884.s003.tif]

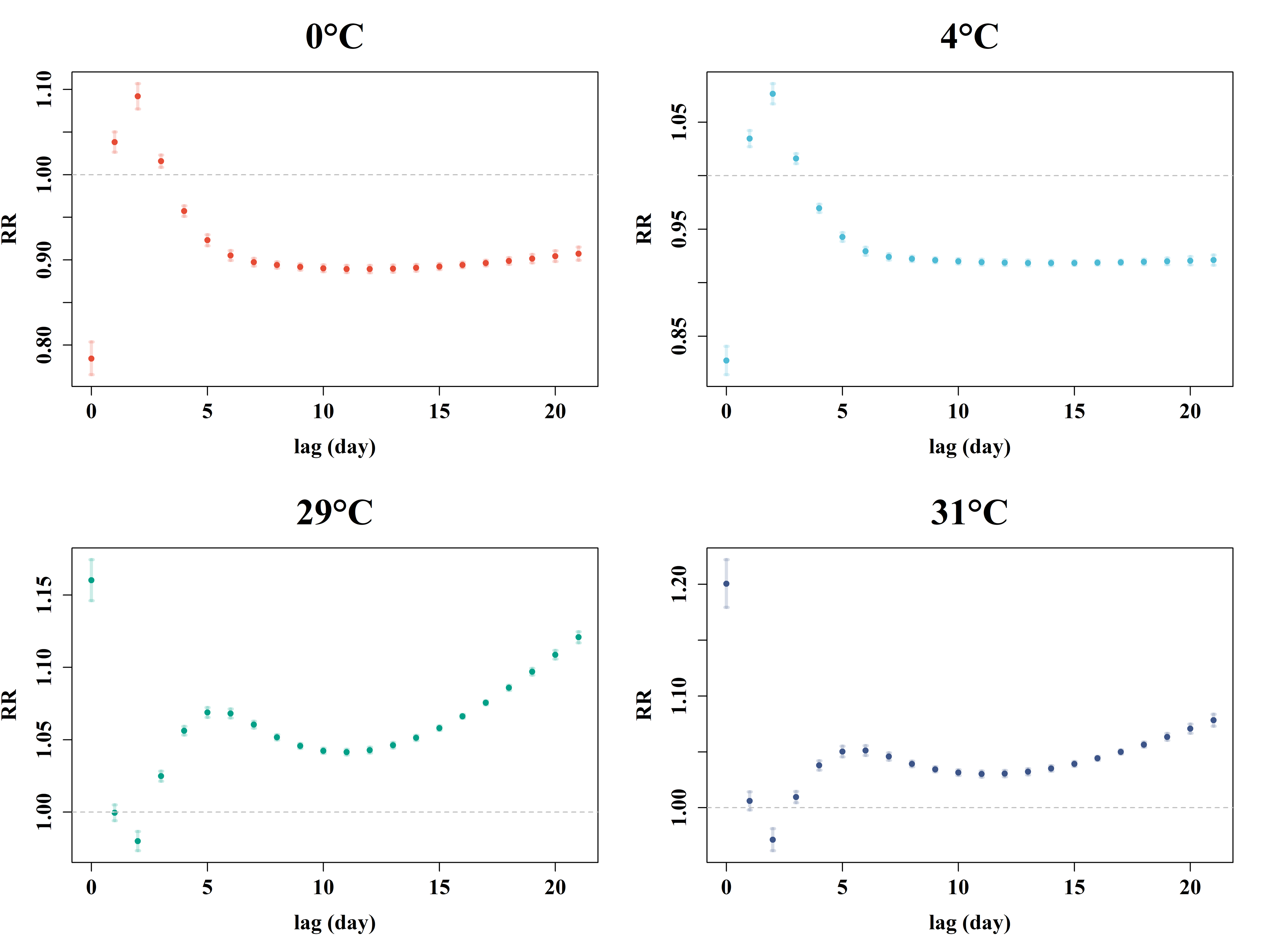

Supplement: S2 Fig — (TIF) [file pntd.0012884.s004.tif]
